# External ventricular drain placement is associated with lower mortality after intracerebral hemorrhage with intraventricular hemorrhage

**DOI:** 10.1186/s12245-022-00450-4

**Published:** 2022-09-15

**Authors:** Andrew D. Warren, Qi Li, Kristin Schwab, Brenna McKaig, Alexa N. Goldstein, Steven M. Greenberg, Anand Viswanathan, Christopher Anderson, M. Edip Gurol, Aman Patel, Joshua N. Goldstein

**Affiliations:** 1 Department of Neurology, Massachusetts General Hospital Stroke Research Center, Harvard Medical School, Boston, MA USA; 2 Department of Neurology, The First Affiliated Hospital of Chongqing Medical University, Chongqing, China; 3 Department of Emergency Medicine, Massachusetts General Hospital, Harvard Medical School, Boston, MA USA; 4 Department of Neurology, Brigham and Women’s Hospital, Harvard Medical School, Boston, MA USA; 5 Department of Neurosurgery, Massachusetts General Hospital, Harvard Medical School, Boston, MA USA

**Keywords:** Cerebral hemorrhage, Intracranial hemorrhages, Ventriculostomy, Brain diseases, Cerebrovascular disorders

## Abstract

**Background and aims:**

Many patients with intracerebral hemorrhage (ICH) develop intraventricular hemorrhage (IVH), which is associated with higher mortality and worse clinical outcome. External ventricular drains (EVDs) are often placed, but there is little data on how much patients benefit from this intervention. We explored the use, timing, and location of EVD in ICH patients and any association with clinical outcome.

**Results:**

During the study period, 2870 patients presented with primary ICH, and 2486 were included in analyses. Overall, patients were 73 (± 13) years old; 54% were male, and 46% had associated IVH. An EVD was placed in 29% of patients with IVH and 4% of those without. IVH patients with EVD were younger (67 ± 13 vs 74 ± 13, *p* < 0.001), had larger IVH volumes (17 mL vs 8 mL, *p* < 0.001), and lower GCS scores (7 vs 10, *p* < 0.001), compared to those without EVD. Ninety-day mortality was available in 2486 (100%) patients, while 90-day mRS was available in 1673 (67.3%). In univariate analysis, EVD placement was associated with lower likelihood of 90-day mortality (53% vs 59%, *p* = 0.048) but higher likelihood of poor outcome (88% vs 85%, *p* < 0.001) in those for whom this was available. Those with poor outcomes underwent faster EVD placement (0.46 days vs. 0.96 days, *p* = 0.01). In multivariate analysis, EVD placement was associated with lower 90-day mortality (*OR* 0.19, 95% *CI* 0.053–0.657, *p* = 0.009), but not with lower odds of poor outcome (*OR* 1.64, 95% *CI* 0.508–5.309, *p* = 0.4). In multivariate analysis, days to EVD placement was associated with lower 90-day mortality (*OR* 0.69, 95% *CI* 0.49–0.96, *p* = 0.027).

**Conclusion:**

IVH is relatively common after ICH. After controlling for potential confounds, EVD placement is associated with lower mortality, but not clearly with better neurologic outcome. In addition, more rapid EVD placement is associated with higher mortality, potentially reflecting early development of herniation or obstructive hydrocephalus.

## Introduction

Spontaneous intracerebral hemorrhage (ICH) accounts for 10–15% of strokes globally and is the deadliest stroke subtype [1–4]. One common complication of ICH is extension of blood into the ventricles, causing associated intraventricular hemorrhage (IVH) in approximately 45% of patients [5].

Patients with ICH with IVH extension are at substantially higher risk of poor outcome [5, 6]. This may be due to thalamus and reticular activating system damage, obstructive hydrocephalus, or inflammatory reactions10. The blood in the ventricles also commonly correlates with increased intracranial pressure (ICP), which may contribute to worse outcome [5].

One common treatment for severe IVH is to insert an extraventricular drain (EVD), which can drain cerebrospinal fluid (CSF) and blood from the ventricles and reduce ICP [7, 8]. The value of EVD has never been shown in a randomized trial, and the evidence base is limited to small observational studies [7, 9, 10]. As a result, the magnitude of any benefit from EVD is not clear, nor whether there is a subset of patients that benefit the most.

We therefore sought to examine EVD use in a large cohort of ICH patients and hypothesized that EVD use would be associated with improved outcome and lower mortality at 90 days. We also explored whether any particular subgroup may benefit.

## Results

**Fig. 1:**
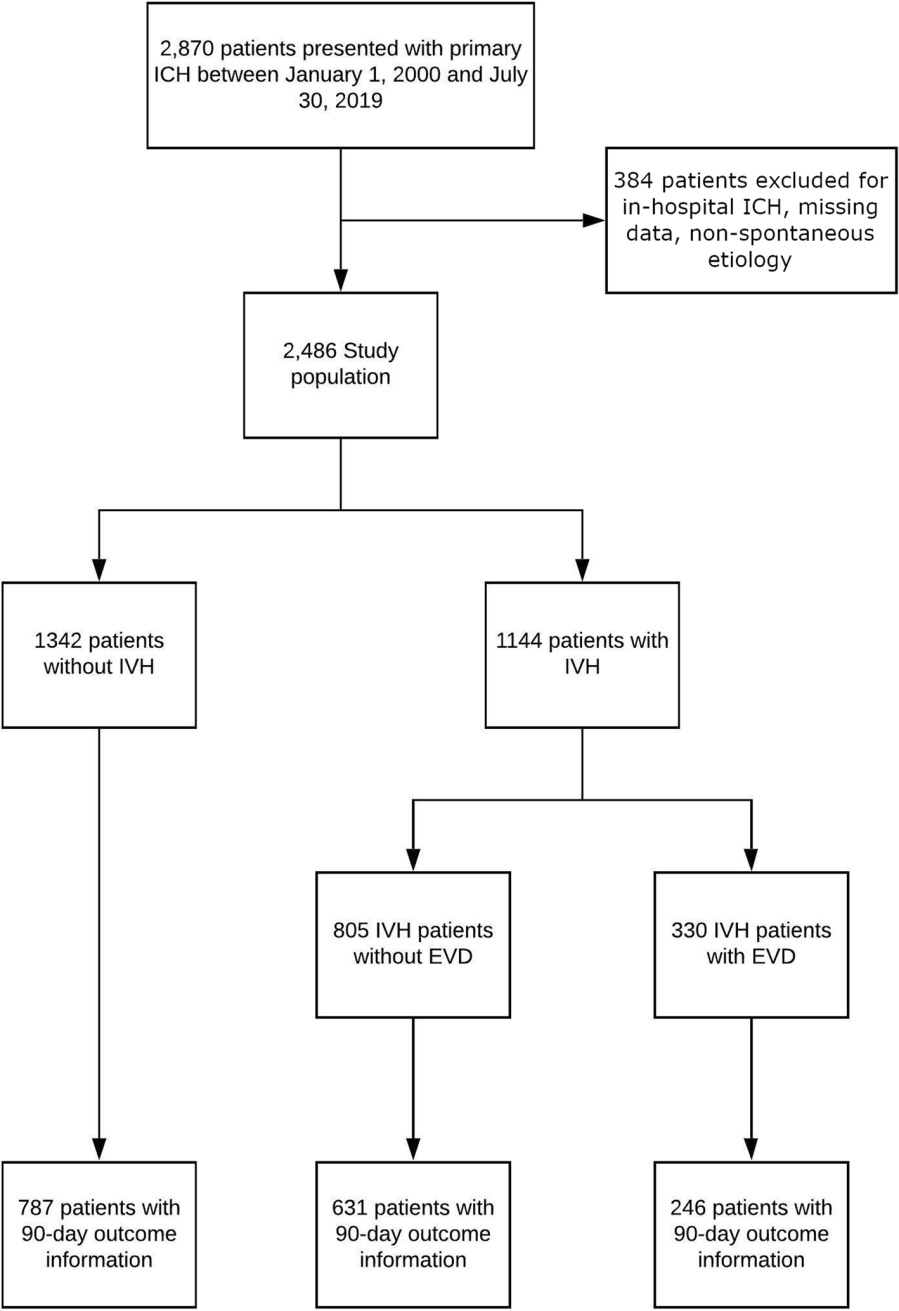
Enrollment flowchart. Abbreviations: EVD, external ventricular drain; ICH, intracerebral hemorrhage; IVH, intraventricular hemorrhage

During the study period, 2870 patients presented with ICH. Three-hundred eighty-four were excluded for inhospital ICH, insufficient data, and possible confounding factors, leaving 2486 for analysis (Fig. 1). Table 1 shows the demographics of this cohort. Overall, patients were 73 + / − 13 years old; 54% were male, and 46% had IVH. An EVD was placed in 29% of patients with IVH and 4% of those without.

**Table 1:** Baseline characteristics of ICH patients with and without ventricular extension

**Variable**	**Without IVH (*n* = 1342)**	**With IVH (*n* = 1144)**	***p*-Value**
**Demographics**			
Age	72 ± 12	72 ± 12	0.3
* Sex*			0.29
% male	53%	55%	
% female	47%	45%	
**Previous medical history**			
ICH	11%	10%	0.16
Ischemic stroke	16%	15%	0.37
Hypertension	79%	84%	0.001
Diabetes	21%	24%	0.043
Hypercholesterolemia	46%	43%	0.105
**ICH characteristics**			
ICH volume median (IQR)	9 (3–24)	29 (10–73)	< 0.001
IVH volume median (IQR)	0 (0–0)	10 (3–26)	< 0.001
* Location*			0.308
Lobar	50%	43%	< 0.001
Deep	37%	48%	< 0.001
Cerebellar	8%	6%	0.009
Brain stem	4%	4%	0.362
**Admission characteristics**			
Pre-stroke mRS 0 or 1	70%	69%	0.795
Initial SBP	171 ± 34	181 ± 50	< 0.001
Anticoagulants	20%	26%	< 0.001
Antiplatelets	44%	45%	0.656
Initial GCS median (IQR)	15 (13–15)	9 (5–14)	< 0.001
**Outcomes**			
Intubated	18%	57%	< 0.001
DNR in first 24 h	14%	33%	< 0.001
CMO in first 24 h	6%	27%	< 0.001
90-day mortality	19%	57%	< 0.001
Discharge mRS 0–3	27%	8%	< 0.001
* 90-day mRS*^a^	(*n* = 787)	(*n* = 886)	< 0.001
0	12.5%	1.7%	
1	16.0%	2.9%	
2	10.0%	3.2%	
3	14.5%	6.4%	
4	10.7%	6.7%	
5	4.3%	5.0%	
6	32.0%	74.2%	
90-day mRS 0–3	53.0%	14.2%	< 0.001
EVD placement	4%	29%	< 0.001
Hematoma evacuation	5%	8%	< 0.001

Data with normal distribution were shown as mean ± SD. Nominal data were displayed with the prevalence. Ordinal data were shown as median (25–75%). Data were organized into sections with titles in bold text

*Abbreviations*: *ICH* Intracerebral hemorrhage, *IVH* Intraventricular hemorrhage, *EVD* Extraventricular drain, *SBP* Systolic blood pressure, *GCS* Glasgow Coma Scale, *DNR* Do not resuscitate, *CMO* Comfort Measures Only

^a^*mRS*, modified Rankin scale score, available for 1673 patients

Table 2 shows the characteristics of patients with IVH, stratified by EVD placement. Of those patients with IVH, EVD placement was more common in those who were younger (age 67 vs 75, *p* < 0.001), had smaller ICH volumes (20 vs 37 cm^3^, *p* < 0.001), were less likely to have lobar placement of ICH (26% vs 50%, *p* < 0.001), had larger IVH volumes (17 vs 8 cm^3^, *p* < 0.001), and had lower initial GCS scores (7 vs 10, *p* < 0.001).

**Table 2:** Baseline characteristics of IVH patients with or without EVD placement

**Variable**	**IVH without EVD (*n* = 805)**	**IVH with EVD (*n* = 330)**	***p*-Value**
**Demographics**			
Age	75 ± 12	67 ± 12	< 0.001
* Sex*			0.4
% male	55%	57%	
% female	45%	43%	
**Previous medical history**			
ICH	11%	6%	0.02
Ischemic stroke	15%	14%	0.8
Hypertension	82%	89%	0.003
Diabetes	24%	27%	0.2
Hypercholesterolemia	42%	44%	0.7
**ICH characteristics**			
ICH volume median (IQR)	37.4 (10.6–86.2)	19.8 (7.8–46.3)	< 0.001
IVH volume median (IQR)	7.8 (2.4–22.6)	17.0 (5.8–36.4)	< 0.001
* Location*			< 0.001
Lobar	50%	26%	< 0.001
Deep	44%	56%	< 0.001
Cerebellar	3%	12%	< 0.001
Brain stem	3%	5%	0.2
**Admission characteristics**			
Pre-stroke mRS 0 or 1	65%	79%	< 0.001
Initial SBP	177 ± 35	190 ± 75	< 0.001
Anticoagulants	24%	29%	0.07
Antiplatelets	47%	41%	0.06
Initial GCS median (IQR)	10 (5–15)	7.5 (4–13)	< 0.001
EVD placement	4%	29%	< 0.001
**Outcomes**			
Intubated	45%	87%	< 0.001
DNR in first 24 h	39%	17%	< 0.001
CMO in first 24 h	34%	10%	< 0.001
90-day mortality	59%	53%	0.048
Discharge mRS 0–3	9.0%	4.0%	0.007
* 90-day MRS*	(*n* = 631)	(*n* = 246)	0.5
0	2.2%	0.4%	
1	3.2%	2.4%	
2	3.6%	1.2%	
3	5.9%	8.1%	
4	5.9%	8.9%	
5	3.8%	8.1%	
6	75.4%	70.7%	
90-day mRS 0–3	14.9%	12.2%	0.3
Hematoma evacuation	5%	14%	< 0.001

Data with normal distribution were shown as mean ± SD. Nominal data were displayed with the prevalence. Ordinal data were shown as median (25–75%)

*Abbreviations*: *ICH* Intracerebral hemorrhage, *IVH* Intraventricular hemorrhage, *EVD* Extraventricular drain, *SBP* Systolic blood pressure, *GCS* Glasgow Coma Scale, *DNR* Do not resuscitate, *CMO* Comfort measures only

For the remainder of the analyses, patients made comfort measures only (CMO) within 24 h (692 patients) were excluded. Tables 3 and 4 show factors associated with neurologic outcome and mortality within patients with IVH. While mRS was available at discharge in 95% of patients, 90-day mRS was only available in 67%. In univariate analysis of those not made CMO, EVD placement in patients with IVH was associated with higher likelihood of poor outcome (88% vs 85%, *p* = 0.04) and lower mortality (53% vs 59%, *p* = 0.003). However, due to concern that this finding may have been confounded by indication (with EVD placement directed towards the highest risk patients), we performed a multivariable analysis to control for disease severity. Tables 5 and 6 show that in patients with both ICH and IVH, EVD placement was independently associated with lower 90-day mortality (*OR* 0.187, 95% *CI* 0.053–0.657, *p* = 0.009) but was not independently associated with lower 90-day poor outcome (*OR* 1.642, 95% *CI* 0.508–5.309, *p* = 0.4). We also examined characteristics of EVD placement such as timing and early hydrocephalus. In multivariate analysis controlling for age, initial ICH and IVH volume, and GCS at admission, longer time to EVD placement was associated with lower 90-day mortality (*OR* 0.687, 95% *CI* 0.49–0.96, *p* = 0.027), and hydrocephalus was positively associated with 90-day mortality (*OR* 1.973, 95% *CI* 1.12–3.47, *p* = 0.018).

**Table 3:** Factors associated with 90-day neurologic outcome

**Variable**	**Good outcome (*n* = 543)**	**Poor outcome (*n* = 1130)**	***p*-Value**
Age	70 ± 12	75 ± 12	< 0.001
Previous ICH	5%	10%	< 0.001
Hypertension	77%	83%	0.001
ICH volume median (IQR)	7 (2–19)	39 (13–81)	< 0.001
IVH volume median (IQR)	0 (0–0)	5.0 (0.0–22.0)	< 0.001
Lobar location	50%	46%	0.096
Deep location	40%	41%	0.673
Cerebellar location	7%	7%	0.735
Anticoagulated	19%	27%	< 0.001
Initial SBP	170 ± 33	180 ± 51	< 0.001
EVD	7%	21%	< 0.001
Intubation	12%	60%	< 0.001

Data with normal distribution were shown as mean ± SD. Nominal data were displayed with the prevalence. Ordinal data were shown as median (25–75%)

Abbreviations: *ICH* Intracerebral hemorrhage, *IVH* Intraventricular hemorrhage, *EVD* Extraventricular drain, *SBP* Systolic blood pressure, *GCS* Glasgow Coma Scale, *DNR* Do not resuscitate, *CMO* Comfort measures only

**Table 4:** Factors associated with 90-day mortality

**Variable**	**No death within 90 days (*n* = 1578)**	**Death within 90 days (*n* = 908)**	***p*-Value**
Age	71 ± 13	76 ± 12	< 0.001
Previous ICH	10%	11%	0.361
Hypertension	80%	83%	0.079
ICH volume	9 (3–21)	48.5 (17–88)	< 0.001
IVH volume	0 (0–1.4)	7.2 (0–26.9)	< 0.001
Lobar location	46%	48%	0.386
Deep location	43%	39%	0.08
Cerebellar location	8%	6%	0.117
Anticoagulated	19%	28%	< 0.001
Initial SBP	174 ± 40	179 ± 47	0.013
EVD	12%	21%	< 0.001
Intubation	19%	66%	< 0.001

Data with normal distribution were shown as mean ± SD. Nominal data were displayed with the prevalence. Ordinal data were shown as median (25–75%)

*Abbreviations*: *ICH* Intracerebral hemorrhage, *IVH* Intraventricular hemorrhage, *EVD* Extraventricular drain, *SBP* Systolic blood pressure

**Table 5:** Multivariate analysis of EVD and mortality among patients with IVH

**Variable**	**OR (95% *CI*)**	***p*-Value**
EVD	0.392 (0.166–0.926)	0.033
Age	1.08 (1.049–1.112)	< 0.001
ICH volume	1.018 (1.008–1.027)	< 0.001
IVH volume	1.022 (1.006–1.038)	0.006
Initial GCS	0.839 (0.771–0.913)	< 0.001

*Abbreviations*: *EVD* Extraventricular drain, *GCS* Glasgow Coma Scale

**Table 6:** Multivariate analysis of EVD and poor outcome among patients with IVH

**Variable**	**OR (95% *CI*)**	***p*-value**
EVD	2.797 (0.951–8.226)	0.062
Age	1.06 (1.022–1.099)	0.002
ICH volume	1.027 (1.01–1.045)	0.002
Lobar hemorrhage	0.166 (0.061–0.451)	< 0.001
Pre-stroke mRS of 0–1	0.172 (0.052–0.563)	0.004
Initial GCS	0.879 (0.779–0.992)	0.037

*Abbreviations*: *EVD* Extraventricular drain, *GCS* Glasgow Coma Scale

**Fig. 2:**
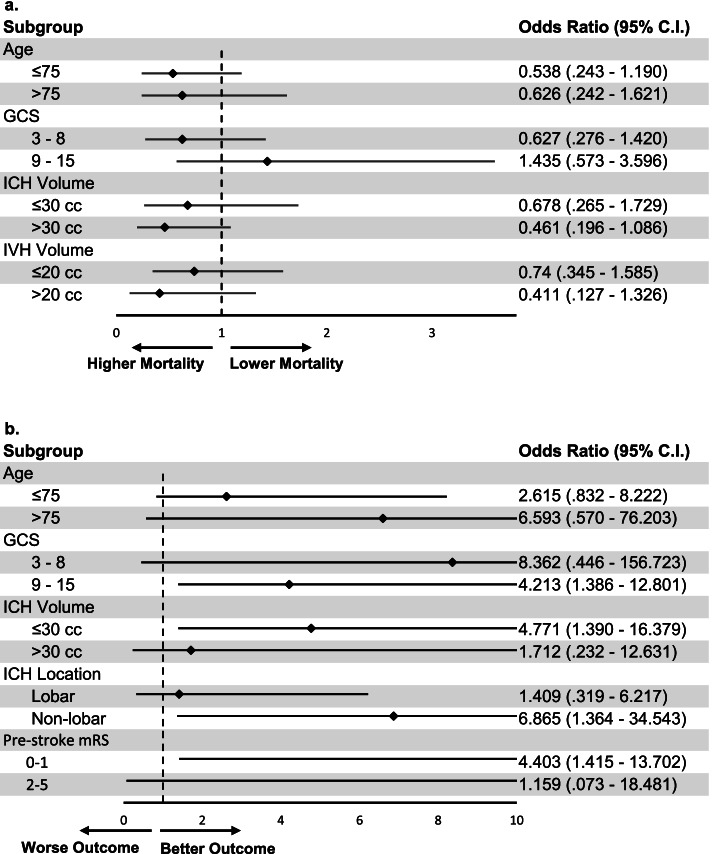
**a** Forest plot of EVD and 90-day mortality: a subgroup analysis. Abbreviations: CI, confidence interval; EVD, external ventricular drain; GCS, Glasgow Coma Scale; ICH, intracerebral hemorrhage; IVH, intraventricular hemorrhage. **b** Forest plot of EVD and 90-day poor outcome: a subgroup analysis.Abbreviations: CI, confidence interval; EVD, external ventricular drain; GCS, Glasgow Coma Scale; ICH, intracerebral hemorrhage; IVH, intraventricular hemorrhage; mRS, modified Rankin scale

In an exploratory analysis, we examined whether EVD placement was associated with disproportionate benefit in any particular subgroup (Fig. 2). We found that EVD placement was associated with disproportionate reduction in mortality in younger patients, patients with greater ICH volumes, patients with smaller IVH volumes, and patients with higher mRS prior to the index event.

## Discussion

Overall, we found that in patients with ICH and IVH, EVD placement was independently associated with lower mortality, but not with better 90-day neurologic outcome. Other factors associated with 90-day mortality and outcome included age, ICH and IVH volume, ICH location, and initial GCS score.

We noted several factors associated with IVH incidence. These included a past medical history of hypertension and diabetes, higher initial systolic blood pressure, larger ICH volume, basal ganglia location, and lower initial GCS score. Other authors have found similar findings [5, 11]; however, some have also found older age to be associated with IVH [5]. We are unaware of prior findings suggesting a linkage between diabetes and IVH. We also confirmed prior findings that IVH predicts poor outcome [9, 12–14].

Few other studies have examined the link between EVD placement and other clinical markers of ICH and outcome. Herrick et al. (2014) found that EVD placement is primarily in patients with greater IVH volumes, younger age, lower admission GCS score, and basal ganglia location [9]. Similarly, we found that EVDs are placed in those with smaller ICH volume, larger IVH volume, basal ganglia location, higher initial blood pressure, and lower initial GCS. Not surprisingly, those with withdrawal of care were less likely to undergo EVD placement.

Our finding that EVD placement was associated with worse neurologic outcome in univariate analysis, but not after multivariate analysis, suggests that its use is preferentially directed towards the most severely injured patients. We note that Nieukamp et al. (2014) reached similar conclusions from their metanalyses of published literature [14]. We can thus infer that some of the findings on univariate analysis may be due to confounding by indication. The fact that controlling for disease severity reverses this finding suggests that clinical providers may be appropriately targeting those patients for EVD placement who are likely to truly benefit. Overall, our data suggest that the current use of EVD in clinical practice is justified and likely lowering mortality.

Whether lowering mortality leads to an increase in “good” neurologic outcome is unclear. Other clinical trials of surgical therapy have found reductions in mortality, without a corresponding improvement in neurologic outcome [11, 15–17], suggesting that some interventions may save lives but with severe associated morbidity, rather than leading to “good” recovery. Another study has similarly found that EVD subjects exhibited lower mortality but worse outcome [18]. It is not clear whether EVD placement falls into this category or whether any benefit from its use comes from bundling it with multiple other interventions.

While previous projects have examined EVD placement and outcome, this analysis includes a substantially larger cohort than prior efforts [7, 9, 10], lending more power to adjust for multiple factors related to clinical outcome. Other papers previously examining EVD usage in large populations have focused on assessing EVD placement and complications but have not looked directly at clinical outcome [5]. We were able to include comparable data from this relatively large population of EVD recipients over a time span of 19 years because EVD placement standards and guidelines for treatment of ICH with IVH at the study center did not change greatly over the study duration. We also investigated variables related to EVD placement such as timing of EVD placement and early presence of hydrocephalus. Hydrocephalus was positively associated with 90-day mortality, meaning that the presence of hydrocephalus on the first CT was correlated with a higher likelihood of death within 90 days. Time to EVD placement was negatively associated with 90-day mortality, meaning that the later an EVD was placed, the more likely patients were to survive past 90 days. This may mean that acute EVD places additional stressors on patients with intracerebral hemorrhages, but it could also indicate that the most severe cases are given priority for EVD placement as a final measure to attempt to improve outcome.

There were several limitations to the scope of this study. First, as this was an observational study, EVD placement was at the direction of clinical providers, and there may have been confounding by indication. Clinicians may have selected those patients most likely to have good outcome to undergo EVD placement. We attempted to control for this with multivariable analysis, but unmeasured confounders may still have been present. Second, we did not directly assess hydrocephalus, the expansion of fluid in the ventricles that is thought to cause many of the problems associated with IVH. We also did not assess the position of EVD placement, the experience of the neurosurgeon involved, or the actual amount of CSF drainage, all of which may have an impact on its efficacy. The EVD placement decision was made by individual clinicians, raising the risk of confounding by indication. Many patients did not have 3-month functional outcome available; as a result, poor outcome may be overrepresented as those who died were more likely to have available outcomes (Social Security Death Index) than those who did not. We were also unable to control for thrombolytic treatments received. Finally, as this was a retrospective single-center study, the results may not be generalizable to other institutions with different patient populations or practice patterns.

## Conclusion

EVD placement appears independently associated with lower mortality after ICH with IVH. Future prospective studies should evaluate which patients benefit most from this intervention.

## Methods

### Study population

We performed a retrospective analysis of a prospectively collected cohort of consecutive patients with primary ICH presenting to a single academic medical center between the years 2000 and 2019 [19, 20]. Patients with secondary ICH including trauma, aneurysm, ischemic stroke, tumor, arteriovenous malformation, or other causes were excluded. Patients without information regarding IVH the presence and EVD placement were also excluded. Demographic and clinical data were collected prospectively. Data on EVD placement were collected via medical record review. Patients consenting to future contact were prospectively assessed via 3-month telephone interviews to determine clinical outcome, and mortality data were collected via serial queries of the Social Security Death Index. Good outcome was defined as 90-day modified Rankin score (mRS) of 0–3. This study was performed with the approval of our institutional review board. Patients provided informed consent, or need for consent was waived for retrospective review.

### Clinical data

Baseline data collection was performed as described previously [19, 20]. Briefly, clinical variables collected included age, sex, medical history of vascular risk factors, current medications, and characteristics upon presentation. Intraventricular thrombolysis was performed very infrequently (< 5 times) and so was not included in the analysis. Do not resuscitate (DNR) orders and comfort measures only (CMO) orders were captured when they occurred within the first 24 h. Time to EVD placement was measured as a whole number of days. Since accurate times were not consistently available in the records, we rounded to the nearest day. Subjects were recorded as intubated if they were intubated at any time during their hospitalization for this event. The enrolling hospital had no formal clinical policy regarding EVD placement. Hydrocephalus was captured if specifically diagnosed by the neuroradiology or clinical teams. Discharge modified Rankin scale (mRS) was calculated on the day of discharge, and follow-up mRS was assessed through follow-up telephone calls 3 months after the index ICH or through records of death before 3 months. Mortality was assessed by periodic review of the Social Security Death Index.

### Neuroimaging acquisition and analysis

For all subjects, the first available CT was used to calculate ICH and IVH volumes. Volumes were read independently using Analyze Direct 11.0 software — a semiautomated computer-assisted technique with excellent interrater reliability [19, 21]. Trained raters were blinded to all clinical information while assessing scans.

### Statistics

Demographics and clinical characteristics were compared in univariate analyses between IVH and non-IVH ICH cases and within IVH patients between EVD and non-EVD patients. Medians between two integer variables were rounded down. Initial multivariable analysis was performed on IVH cases who were not made CMO during their course. The model included variables identified as significant and near significant in univariate analysis (*p* < 0.1). Highly collinear variables were removed from multivariable analysis. These included intubation, the presence of DNR status within 24 h, and the presence of CMO status within 24 h. Remaining variables were narrowed down via backwards stepwise logistic regression, with mortality and 90-day poor outcome as dependent variables. The SPSS 24 statistical package was used for statistical analysis (IBM Corp., Armonk, NY, USA). Significance level was set at 0.05 for all analyses.

## Data Availability

The datasets generated and/or analyzed during the current study are not publicly available due to protected health information, but deidentified data are available from the corresponding author on reasonable request.
